# Pregnancy, Labor, and the Puerperal State

**Published:** 1896-09

**Authors:** 


**Pregnancy, Labor, and the Puerperal State.** By EGBERT H. GRANDIN, M.D., and GEORGE W. JARMAN, M.D. Pp. viii., 261. Philadelphia: The F. A. Davis Company. London: F. J. Rebman. 1895.

In December, 1895, we reviewed *Obstetric Surgery*, a work by the authors of this volume, which, however, regards midwifery, as much as possible, from a medical point of view. Now, in the art and science of obstetrics, the practice of medicine and of surgery are so intimately connected together, that any attempted divorce of this kind is both difficult to effect and not particularly happy in the results which follow. The same remark holds good with regard to other sciences, which in such a comprehensive branch of general practice as midwifery are closely intermingled, such, for instance, as biology, embryology, physiology, pathology, &c. They cannot be dissociated and dislocated from one another, but must be allowed to remain together as one harmonious whole. Upon this principle all our standard and classical works on Midwifery, both old and new, have been composed, and we do not wish to see so good a plan deranged, at least in those which are primarily intended as text-books for students.

Like its predecessor, the present work is exceedingly well got up, equally as regards paper, type, and illustrations.

Although the graphic art is much more sparingly employed than in *Obstetric Surgery*, the authors consider this feature of the book to be one of its chief merits, if we may judge from the following remark in the preface:—" In the matter of illustration, fidelity to nature has been the aim rather than attempt at artistic effect. It has not been deemed advisable to insert the numerous wood-cuts which from time immemorial have been copied from one work to another, since the majority teach nothing which cannot be learned to better advantage at the bedside,—indeed, which can only be properly learned there." Unfortunately the "New Photography" has not yet rendered the human body so transparent that we can see at a glance the various malpresentations of the fœtus *in utero*; but are still obliged to find them out by palpation at the bedside. The often copied woodcuts and diagrams convey more exact ideas to a student's mind than any that can be derived from photographs of the external parts of the woman's body. These it would have been better to have omitted, as well as the ridiculous-looking costume in which she herself and often a multiplicity of attendants are enveloped. For instance, there are stiff, straight figures of a pregnant woman, like a corpse wrapped in a winding-sheet, which covers every part of her body except the enlarged abdomen and breasts. Another photograph purporting to represent the coloured areola of the breast in pregnancy, which is represented by a monotonous shade of brown like "burnt umber," is vastly inferior to the life-like tints of nature so faithfully portrayed in Montgomery's classical work, the *Signs of Pregnancy*. In some other photographs representing the phenomena of labour, very little of the parturient woman is seen, except the parts immediately concerned in the process; all the rest of her is hidden by white winding-sheets, and as many as five attendants are around her clad in costumes like those worn by French cooks, and suggesting the idea that the "broth is likely to be spoilt by too many cooks." Such is the amount of knowledge which the student would be likely to gain from representations in every respect inferior to the much despised outline drawings and diagrams by which the "men-midwives" of old used to impart knowledge to their pupils. As the authors of this volume justly remark, the signs of pregnancy and commencing labour can only be learned at the bedside. In learning midwifery, we gain next to nothing from the sight and still less from photographic plates. In fact these, upon which the authors pride themselves, seem to us a failure as regards teaching, and, as regards appearance, even less ornamental than they are useful. We are glad, however, to mention a few exceptions to this rule, such, for instance, as Plates XII., XIII., and XIV., representing the fœtal skull, and which are only inferior to the dried crania from which they are taken.

The general arrangement of the work on the whole is good, and the directions especially as to treatment are well and clearly expressed. The treatment, however, on the whole is too medical, and, as we remarked before, too much separated from surgery. When any operation is needed we are at once referred by the authors to their *Obstetric Surgery*. The book is divided into three parts: (I.) Pregnancy, normal and abnormal; (II.) Labour, natural and unnatural, including also the care of the new-born infant; (III.) The Puerperal State, both normal and pathological. Now, although the treatment adopted in abnormal cases is as purely medical as possible, the authors have no objection to using the strongest and most "heroic" remedies, both internal and external, especially in cases of septicæmia. For instance, we read in page 231: "If the site of infection is a perineal wound which has been sutured, the stitches should at once be removed, the infected surface washed off with bichloride solution 1 to 5000, and then cauterized with nitrate-of-silver solution 60 grains to the ounce." A number of milder remedies are then recommended to follow these. In that very rare but dangerous complication of labour, inversion of the uterus, we are told that should all attempts to reduce the inversion fail, "the sole resource—desperate as it is—is to open the abdomen and stretch the contraction ring." Thus obstetric surgery is called in as a last resource; but verily its tender mercies are cruel after those of obstetric medicine!

The only further remark we have to offer is, that if *Obstetric Surgery* and *Pregnancy, Labor, and the Puerperal State* had been judiciously blended together, omitting a number of showy but nearly useless photographs, and substituting for them some simple woodcuts, a good practical volume on Midwifery, at a very moderate cost, would have been the result.

